# SHAPING PREFERENCES FOR AGING-SPECIFIC CONTINUING EDUCATION BY LICENSED PROFESSIONAL COUNSELORS

**DOI:** 10.1093/geroni/igac059.1088

**Published:** 2022-12-20

**Authors:** Nicholas Schmidt, Ann Steffen

**Affiliations:** Boston VA Healthcare System, Boston, Massachusetts, United States; University of Missouri-St. Louis, St. Louis, Missouri, United States

## Abstract

Although the prevalence of depression, like many other mental health disorders, declines with age, older adults who seek behavioral health services often present with depressive symptoms mixed with cognitive complaints. This creates challenges for generalist practitioners, who are responsible for the majority of mental health care of aging individuals. Ageism and misconceptions about older adults are theorized barriers to clinicians seeking aging-specific specialty training. This completed experimental study showed that assignment to receiving foundational information about aging, including debunking common misconceptions about cognitive aging, influenced choice of continuing education (CE) preferences of generalist Licensed Professional Counselors (LPCs). Among the randomly generated pool of LPCs (N=120), participants who received aging-specific information were more likely to choose an aging-specific CE option, F(4, 107) = 5.35, p<.001. Demographic variables, perceived competence for working with older adults, knowledge of aging, and ageist beliefs were also collected; analyses including these variables will also be presented.

